# Expression patterns of cardiac aging in *Drosophila*

**DOI:** 10.1111/acel.12559

**Published:** 2017-01-16

**Authors:** Leah Cannon, Alexander C. Zambon, Anthony Cammarato, Zhi Zhang, Georg Vogler, Matthew Munoz, Erika Taylor, Jérôme Cartry, Sanford I. Bernstein, Simon Melov, Rolf Bodmer

**Affiliations:** ^1^ Development, Aging and Regeneration ProgramSanford‐Burnham‐Prebys Medical Discovery InstituteLa JollaCAUSA; ^2^ Department of Biopharmaceutical SciencesKeck Graduate InstituteClaremontCAUSA; ^3^ Division of CardiologyDepartment of MedicineJohns Hopkins UniversityBaltimoreMDUSA; ^4^ Department of Biology, Molecular Biology Institute, and The Heart InstituteSan Diego State UniversitySan DiegoCAUSA; ^5^ Buck Institute for Research on AgingNovatoCAUSA

**Keywords:** heart, arrhythmia, cardiomyopathy, senescence, nanofluidics, Odd‐skipped, LysX, MMP1

## Abstract

Aging causes cardiac dysfunction, often leading to heart failure and death. The molecular basis of age‐associated changes in cardiac structure and function is largely unknown. The fruit fly, *Drosophila melanogaster*, is well‐suited to investigate the genetics of cardiac aging. Flies age rapidly over the course of weeks, benefit from many tools to easily manipulate their genome, and their heart has significant genetic and phenotypic similarities to the human heart. Here, we performed a cardiac‐specific gene expression study on aging *Drosophila* and carried out a comparative meta‐analysis with published rodent data. Pathway level transcriptome comparisons suggest that age‐related, extra‐cellular matrix remodeling and alterations in mitochondrial metabolism, protein handling, and contractile functions are conserved between *Drosophila* and rodent hearts. However, expression of only a few individual genes similarly changed over time between and even within species. We also examined gene expression in single fly hearts and found significant variability as has been reported in rodents. We propose that individuals may arrive at similar cardiac aging phenotypes via dissimilar transcriptional changes, including those in transcription factors and micro‐RNAs. Finally, our data suggest the transcription factor Odd‐skipped, which is essential for normal heart development, is also a crucial regulator of cardiac aging.

## Introduction

Aging results in significant deterioration in the structure and function of cardiac muscle. Moreover, heart disease, the primary cause of mortality worldwide, significantly increases with age (Minino *et al*., 2011). However, the molecular basis of cardiac aging (and aging in general) is still far from understood. Therefore, it is imperative to further investigate the molecular changes associated with the aging heart to develop improved diagnostics and therapeutics that prolong cardiovascular health.

It is difficult to study the molecular‐genetic contributions to aging in humans as people have a long lifespan, and there is significant genetic, epigenetic, and environmental diversity among individuals and between populations (Balaresque *et al*., 2007; Lam *et al*., 2012). To date, human studies have uncovered only a few individual genes that show a robust association with aging in the heart (Jeck *et al*., 2012). However, human and mouse studies across a range of tissues have shown age‐related up‐regulation in extracellular matrix and inflammatory response genes, and down‐regulation of ribosomal and mitochondrial genes (Zahn *et al*., 2006, 2007).

The fruit fly, *Drosophila melanogaster,* and the worm, *Caenorhabditis elegans*, provide significant practical advantages over other model systems to study the molecular mechanisms of aging: a short lifespan, low genetic redundancy compared with mammals, and a plethora of tools available to easily manipulate gene expression in a temporal and tissue‐specific manner. In fact, these two model organisms were instrumental in uncovering the first molecular pathways (including insulin and TOR signaling) associated with an increased lifespan in many species (Kenyon *et al*., 1993; Beckman, 2004). However, *Drosophila,* unlike *C. elegans,* has a functional heart with features that are remarkably conserved between flies and humans (Cammarato *et al*., 2011).

The fly heart also shows similar age‐related physiological decline to the human heart. The incidence of cardiac arrhythmias increases with age in humans, and is often caused by ion channel dysfunction (Strait & Lakatta, 2012). Old (5–7 weeks of age) fly hearts are also significantly more arrhythmic and have altered ion channel expression and function compared with young (1‐week old) fly hearts (Ocorr *et al*., 2007b). Humans show exercise intolerance with age, and have a lower maximal heart rate with each decade of life (Strait & Lakatta, 2012). Likewise, old flies have a lower maximal heart rate and decreased tolerance to cardiovascular stress caused by electrical pacing or increased ambient temperature (Paternostro *et al*., 2001; Wessells *et al*., 2004). Aging human hearts stiffen and show relaxation deficits and contractile dysfunction (Strait & Lakatta, 2012). Fly hearts also exhibit elevated stiffness, decreased diastolic diameter, lower fractional shortening and impaired relaxation kinetics with age (Cammarato *et al*., 2008; Fink *et al*., 2009; Kaushik *et al*., 2012, 2015). These changes are the hallmarks of diastolic dysfunction. The phenotypic similarities, coupled with the genetic tractability of the fly, make it an ideal model for gene discovery during cardiac aging and pathogenesis.

The dramatic morphological and physiological differences observed between young and aged adult *Drosophila* myocardium must be accompanied by profound underlying changes in the cardiac transcriptome. Moreover, it is likely that many of the genes responsible for cardiac aging in *Drosophila* also contribute to age‐related heart disease in humans. This is because a large majority of human disease‐causing genes have counterparts in *Drosophila* (Bier & Bodmer, 2004) and its heart is characterized by a cardiac proteome that is very similar to that of vertebrate hearts (Cammarato *et al*., 2011).

To further elucidate the transcriptional changes that occur in the aging heart, we profiled the transcriptomes of isolated cardiac tissue from young (1 week) and old (5 week) flies in two different genetic backgrounds. Bioinformatic enrichment analysis was used to search for micro‐RNAs (miRs) and pathways that contribute to the aging‐dependent changes in the fly heart transcriptome. Additionally, both gene and pathway level meta‐analysis were conducted using published transcriptomic data from aged fly, mouse, and rat hearts to determine the extent of conservation (either at the gene or at the pathway level) between or within species. As these comparisons highlighted significant variability in gene expression, we also used nanofluidic qPCR to examine transcriptional variability between single fly hearts. Finally, *in vivo* functional studies suggested that a key transcriptional circuit involving the transcriptional regulator Odd‐skipped can induce phenotypic changes in aged *Drosophila* hearts.

## Results

### The fly heart ages by known aging mechanisms

**Figure 1:**
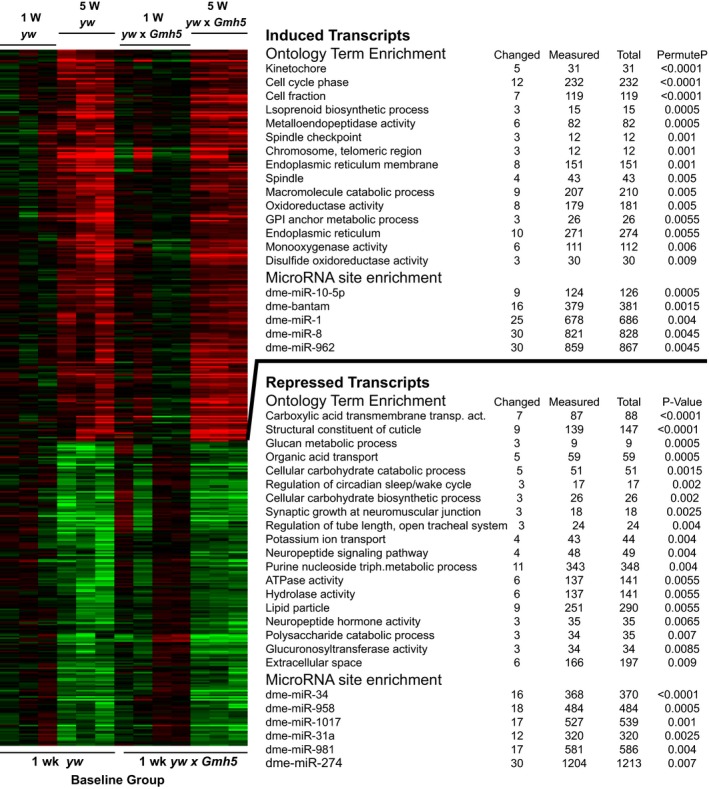
Heatmap of age‐related gene changes in the *Drosophila* heart with associated Gene Ontology terms and miR binding site enrichment. 5‐week‐old *yw* and *yw x GMH5* fly hearts show significant up‐ and down‐regulation of 260 and 202 transcripts (corresponding with 239 and 193 unique genes) respectively compared with 1‐week‐old genotype matched controls (aging *P*‐value < 0.005, interaction *P*‐value > 0.05).

To understand how the fly heart ages on a molecular level, and how this compares with mammalian cardiac aging, we first catalogued the age‐related transcriptional changes in *Drosophila* myocardium and its associated pericardial cells. Microarray analysis was performed to profile global gene expression in the hearts of 1‐ and 5‐week‐old flies. Two different fly background strains were first compared: *yw* and the progeny of the outcross of *yw* to a cardiac Gal4 driver line (see Materials and methods). Results showed 260 and 202 transcripts (corresponding with 239 and 193 unique genes) were significantly up‐ or down‐regulated, respectively, in old fly hearts of both genetic backgrounds compared with genotype‐matched young fly hearts (aging *P*‐value < 0.005, interaction *P*‐value > 0.05, Fig. 1, Table S1, Supporting information; see Methods for statistical analysis). These age‐related transcriptional changes are considered robust as they occur in both genetic backgrounds.

To further probe the molecular mechanisms that likely contribute to cardiac aging, we performed gene ontology (GO) analysis on the genes that were differentially expressed with age in the fly heart. A number of ontological cellular processes known to be involved with mammalian aging were likewise enriched in the aging fly heart gene set (Fig. 1, Table S2, Supporting information).

Firstly, extracellular matrix genes, and in particular metalloproteases, were up‐regulated in aged fly hearts (Fig. 1, Tables S1 and S2, Supporting information). These genes include *Matrix Metalloprotease 1* and *2* (*Mmp1* and *Mmp2*), *neprilysin 2* (*Nep2*), and *TweedleF* (*TwdlF*). Metalloproteases, which regulate extracellular matrix proteins, are similarly altered in the aged mammalian heart and aorta (Muller & Dhalla, 2012; Zhou *et al*., 2012).

DNA replication and repair mechanisms were also up‐regulated in the aged fly heart (Fig. 1), consistent with increased expression of DNA repair genes reported in hearts of aged compared with young mice (Szczesny *et al*., 2010). Several mitochondrial metabolic processes, including ATP synthesis and β‐oxidation decline specifically in aged hearts (Moslehi *et al*., 2012). Consistently, carbohydrate metabolism was down‐regulated in our aged fly hearts (Fig. 1). These findings are suggestive of conserved core mechanisms of cardiac aging from flies to mammals.

### Comparison of aging mechanisms in fly and mammalian hearts

**Figure 2:**
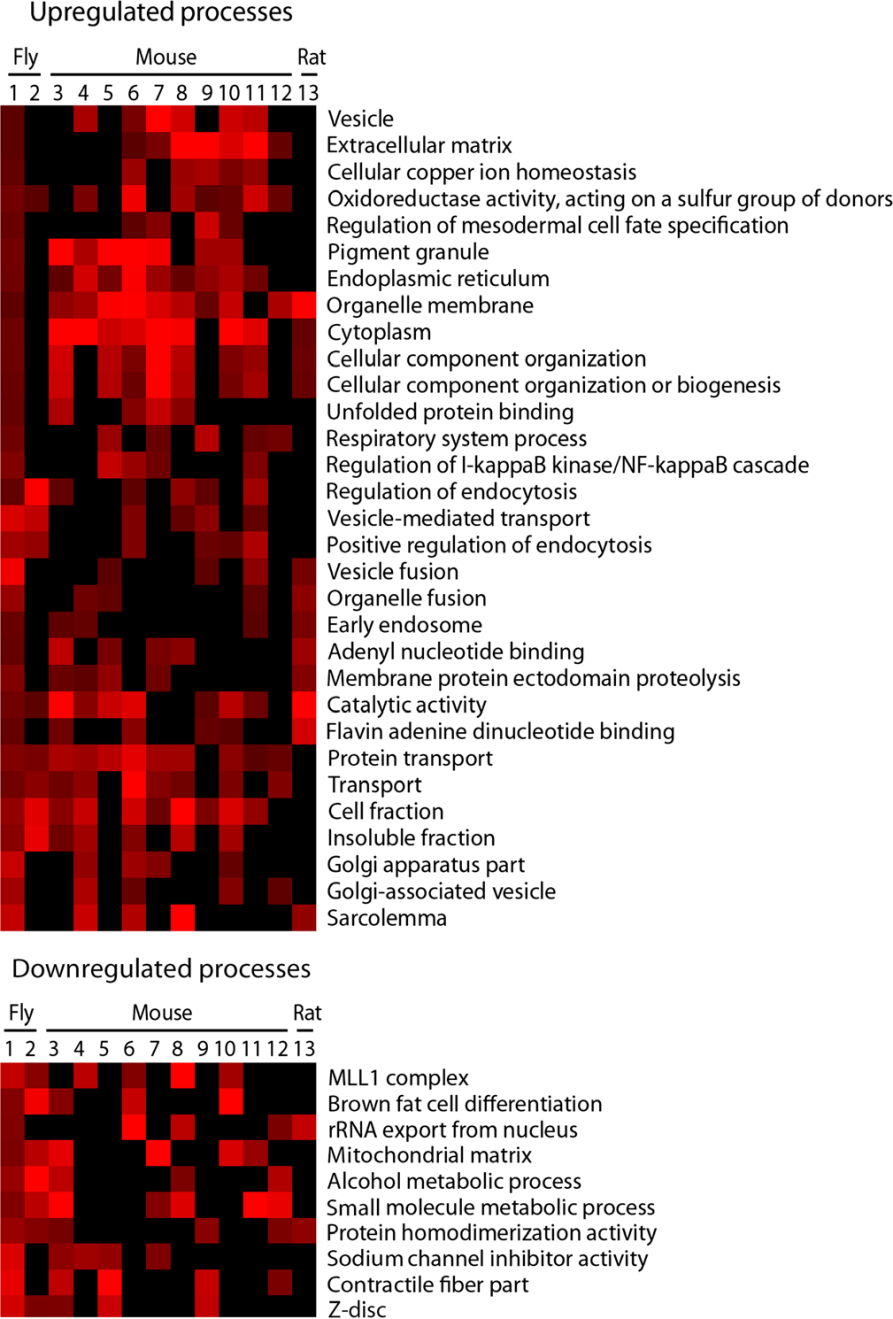
GO terms enriched in up‐ and down‐regulated fly and rodent aging heart gene sets. Red indicates a significantly enriched process (Z>2); brighter color represents a higher Z score. Black indicates no significant enrichment (Z<2).

We examined whether the changes in extracellular matrix, DNA replication and repair mechanisms, mitochondrial metabolism, and other pathways identified in Fig. 1 also occurred in different genetic backgrounds, and different species. To this end, we determined which of the pathways that were altered by aging in our *Drosophila* strains (720 upregulated and 763 downregulated genes‐Aging *P* < 0.05, Interaction *P* > 0.05 |Fold|>20%) were also significantly enriched in at least 4 or more of the 11 published aging rodent heart Affymetrix microarray datasets (10 from mouse hearts and one from rat heart, Table S3, Supporting information; Fig. 2). Also included in this comparison were pathways enriched in genes altered by aging in fly hearts of the *w* ^*1118*^ genetic background (Monnier *et al*., 2012).

Interestingly no pathways were found to be enriched among all cardiac aging datasets. However, protein transport was found to be enriched in the upregulated genes in 10 of the 13 datasets analyzed. This was followed by extracellular matrix and oxidoreductase activity, which were significantly enriched in 8 of the 10 mouse datasets (Fig. 2), but not in the previous study using *w* ^*1118*^ flies (Monnier *et al*., 2012). DNA replication and repair, on the other hand, were not enriched across multiple datasets. Mitochondrial matrix and metabolism terms were enriched in the down‐regulated genes of both fly datasets and four to five mouse datasets, respectively. Additionally, up‐regulation of expression of protein packaging and transport genes, and down‐regulation of contractile function genes were also found in flies and in mice (Fig. 2). This suggests that, while genetic background significantly affects transcriptional changes and the related pathways, there are some genes, such as those associated with the extracellular matrix (remodeling), mitochondrial metabolism and protein handling, which are regulated more consistently across different species during cardiac aging.

### Comparison of genes dysregulated by age in *Drosophila* vs. those of rodent hearts

We next assessed whether age‐associated changes at the individual gene level are also conserved within and between species. Meta‐analysis of rodent (10 mouse and one rat) aging heart datasets showed that expression of 3–18% of genes was altered with age. On average, 10% of genes were over‐expressed and 10% were under‐expressed in old (compared with young) hearts. Expression of 13,249 out of 22,385 genes was significantly altered (corrected *P*‐value < 0.05) in at least one dataset. We focused on conserved genes that displayed transcriptional changes in five or more datasets. With this cutoff, 183 genes and 42 gene orthologues were up‐ or down‐regulated with age, respectively, in the rodent heart data sets (Table S3, Supporting information). There was striking heterogeneity among the mouse datasets with only five genes commonly up‐regulated (Src kinase associated phosphoprotein 2 (*Skap2)*, Amylase 1 (*Amy1)*, Cyclin D1 (*Ccnd1)* and Chemokine (C‐C motif) ligand 8 (*Ccl8*)), and no genes commonly down‐regulated in all 10 datasets.

Next our *yw* fly heart microarray data were compared with the published *w* ^*1118*^ fly data (Monnier *et al*., 2012), using filters of an aging *P*‐value < 0.05 and an interaction *P*‐value < 0.05. This analysis highlighted 720 up‐regulated and 763 down‐regulated aging probe sets in our dataset, compared with 1107 age‐regulated genes in the *w* ^*1118*^ fly data set. Interestingly, the Monnier *et al*. (2012) *Drosophila* study implicated JNK/dJun and Vri/dNFIL3 as major regulators of cardiac senescence. Overall, transcription of 87 genes (including *Mmp1*) was altered in both fly datasets (Table S3, Supporting information). In contrast, *Synaptotagmin VII* (*Syt7*) was the only gene that exhibited significantly altered expression levels in both aged fly and rodent datasets. These data highlight remarkable variability in how hearts age at the individual gene level, even within the same species.

### Transcriptional variability in individual fly hearts

As fly hearts comprise only about 100 cells, 30 hearts were pooled to extract sufficient RNA to perform microarray studies. Recent advances in nanofluidic qPCR methods now allow analysis of RNA from a single cell. To further investigate the transcriptional variability in aging fly hearts, we used Fluidigm's Biomark nanofluidic qPCR to measure gene expression in individual *yw* hearts at 1 and 5 weeks of age. We found up to 16‐ or 32‐fold differences in expression between single hearts (difference of 5–6 PCR cycle thresholds). This expression variability was evident after normalization to traditional housekeeper genes, such as *Acta42* and *Gapdh1* as well as to non‐housekeeper genes such as *Odd* (Fig. S1, Supporting information), and was similar to the transcriptional variation seen in mouse hearts (Bahar *et al*., 2006). Despite the significant variability in gene expression in both fly and rodent hearts, our pathway analysis highlights central conserved cardiac aging mechanisms at the pathway level. Therefore, we next assessed whether alterations in regulatory molecules such as transcription factors and miRs could be driving the conserved aging mechanisms in heart cells.

### The transcription factor Odd‐skipped affects cardiac aging

Transcription factors can regulate expression of suites of genes in various physiological and disease states, and may alter expression of different genes in each individual to arrive at a similar aging phenotype. A number of transcription factors, including *Odd‐skipped* (*Odd*), were found to be up‐regulated with age in the fly heart (Table S1, Supporting information, *Odd* was up 2.7‐fold).

**Figure 3:**
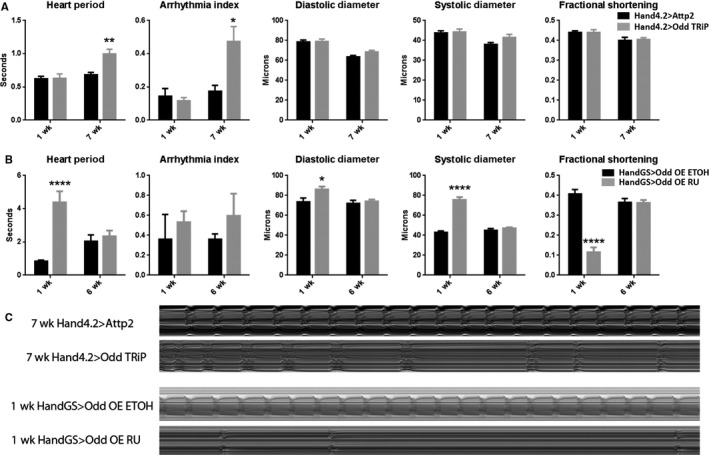
Effect of modulating Odd‐skipped on heart function. (A) Hand4.2>*Odd* TRiP RNAi (grey) compared with control (black) at 1 and 7 weeks by two‐way ANOVA and Tukey's multiple comparison (**P = 0.01, *P < 0.05, *n* = 15–26). Heart Period and Arrhythmia Index exhibit a significant increase with age upon cardiac *Odd* RNAi knockdown. (B) Conditional cardiac overexpression (OE) with HandGS>*Odd*‐OE RU (grey) compared with control (black) at 1 and 6 weeks by two‐way ANOVA and Tukey's multiple comparison (*n* = 15–20, *P < 0.05, ****P < 0.0001). Cardiac *Odd* OE at young but not old age causes increased Heart Period, dilation and reduced contractility (decreased Fractional Shortening). (C) Representative M modes of control and Hand4.2>*Odd* RNAi hearts at 7 weeks and control and HandGS>*Odd*‐OE RU hearts at 1 week.

To see whether Odd may contribute to cardiac aging, *Odd* expression in the fly heart was genetically manipulated and the physiological effects were assessed. RNAi‐mediated *Odd* knockdown with the cardiac‐specific Hand4.2‐Gal4 driver (*Odd* RNAi) caused a slower heart rate (i.e. increased heart period) and significantly increased arrhythmia in old flies, (Fig. 3A,C). Such changes also are known to occur with aging in human hearts (Strait & Lakatta, 2012). Conversely, diastolic diameter and fractional shortening were not affected in *Odd* RNAi fly hearts (Fig. 3A,C). These results, which show aggravated heart dysfunction with age upon *Odd* knockdown, are consistent with increased expression of *Odd* in old hearts to possibly counteract the decline in heart function that normally occurs with age.

As *Odd* over‐expression using the Hand4.2‐Gal4 driver was developmentally lethal, with over 75% of flies dying before eclosion, we used the inducible HandGS‐Gal4 driver to over‐express *Odd* only in adult flies. *Odd* over‐expression for the first week of adult life caused significant bradycardia, systolic and diastolic dilation, and contractile dysfunction as seen by decreased fractional shortening (Fig. 3B,C). However, *Odd* over‐expression for 1–2 weeks in old flies (5–6 weeks of age) did not have a deleterious effect on heart function (Fig. 3B,C). To verify that *Odd* was indeed overexpressed in these old hearts, we directly quantified the amount of *Odd* mRNA, which showed significantly elevated levels (Fig. S2, Supporting information). These findings are consistent with the interpretation that the increased levels of cardiac *Odd* normally seen with age are not pathogenic, but may rather protect from further decline. This suggests that Odd levels are likely to be tightly regulated throughout life, and while *Odd* up‐regulation in old flies may be a compensatory mechanism, overexpression in young fly hearts is detrimental.

### Bioinformatics prediction of Odd target genes in the *Drosophila* heart

**Figure 4:**
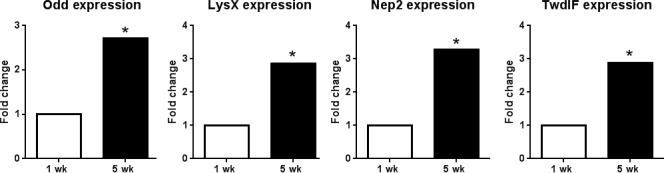
Nanofluidic qPCR results of bioinformatically predicted Odd target genes. mRNA of Odd target genes *Odd*,* LysX*,* Nep2* and *TwdlF* are significantly up‐regulated in 5 week hearts compared with 1 week controls (*n* = 15–16, permutation test < 0.05).

To further understand signaling pathways potentially targeted by Odd or by other transcription factors in the *Drosophila* heart, enrichment analysis was conducted to determine evolutionarily conserved transcription factor binding sites in the proximal promoters of genes either up‐ (*n* = 260) or down‐regulated (*n* = 202) with age (Ho Sui *et al*., 2007). In total, 233 transcription factor binding sites were assessed for statistical enrichment within 2 kb up‐ and downstream of the transcription start site and ranked by significance score. Odd binding sites were the most significantly enriched sites (*Z* score = 9.4; *P* < 1e^−5^) in the upregulated genes (Table S4, Supporting information). Conversely, Odd sites were not significantly enriched (*Z* = −1.2) in the promoters of downregulated genes suggesting that Odd is acting primarily as a transcriptional activator in aged fly hearts. Several predicted Odd target genes, including the antimicrobial peptide *Lysozyme X* (*LysX*), and the extracellular matrix proteins *Neprilysin2* (*Nep2*) and *TweedleF* (*TwdlF*), were found by nanofluidic qPCR to be up‐regulated with age in the fly heart (Fig. 4), suggesting that transcriptional regulation of these genes may, in part, be regulated by Odd.

### LysX expression affects cardiac aging

**Figure 5:**
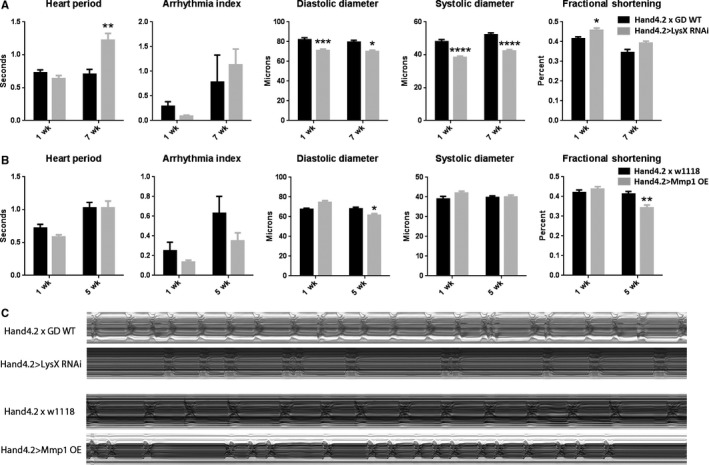
Effect of modulating LysX or Mmp1 on heart function. (A) Hand4.2>*LysX RNAi* (grey bars) compared with control (black bars). Comparisons analyzed by two‐way ANOVA and Tukey multiple comparisons (*P < 0.05, **P < 0.01, ***P < 0.001, ****P < 0.0001; *n* = 8–20). (B) Hand4.2>*Mmp1‐OE* (grey) compared with control (black) at 1 and 5 weeks. Comparisons analyzed by two‐way ANOVA and Tukey multiple comparisons (*n* = 15–30, *P < 0.05). (C) Representative M modes of control vs. Hand4.2>*LysX RNAi* at 7 weeks and control vs. Hand4.2>*Mmp1‐OE* at 5 weeks.

LysX is the fly orthologue of the human antimicrobial peptide lysozyme. RNAi‐mediated cardiac‐specific *LysX* knockdown caused diastolic and systolic constriction in both young (1 week) and old (7 week) hearts and bradycardia in old flies (Fig. 5A,C). The bradycardia seen in LysX‐deficient hearts phenocopies that seen in Odd‐deficient hearts. Although it is well known that microbial load increases with age in the heart, it is not clear whether such elevated loads influence cardiac function. The findings that *LysX* is upregulated with age and that *LysX* knockdown negatively affects the aging heart are consistent with the hypothesis that *LysX* may have a protective role, especially at old age.

### *miR‐1* levels are reduced in aging *Drosophila* hearts

miRs are small non‐coding RNAs that can target numerous mRNAs for degradation or translational repression. A GO Elite (Zambon *et al*., 2012) search for miRs revealed that the genes up‐regulated with age in the fly heart contain a significant enrichment of binding sites for several miRs, including for *miR‐1* (Fig. 1). Furthermore, we found *miR‐1* is abundantly expressed in the adult *Drosophila* heart, just as it is in human and other mammalian hearts (Zhao *et al*., 2005), and is significantly down‐regulated with age (Fig. S3, Supporting information). These data support our informatics prediction and suggest that *miR‐1* levels may contribute to cardiac aging by differentially modulating target gene expression.

### Mmp1 expression affects cardiac aging

*Drosophila matrix metalloprotease 1* (*Mmp1*), the homolog of a number of mammalian *Mmps*, is up‐regulated in old fly hearts (Fig. 1) and has both an Odd consensus sequence and *miR‐1* binding site. Matrix metalloproteases (Mmps) are important players in tissue remodeling as they regulate extracellular matrix, mitochondrial, sarcomeric, and cytoskeletal proteins (Ali *et al*., 2011). Thus, metalloproteases may also be important in age‐related cardiac decline. However, the molecular signaling mechanism that activates these proteases and their subsequent effects in the aging heart are not well understood. Although cardiac knockdown of *Mmp1* had no effect, over‐expression (*Mmp1‐OE*) in the fly heart caused a similar phenotype to *Odd‐OE* at young ages, i.e. a reduction in fractional shortening (compare Fig. 5B,C to Fig 3B,C). These data are consistent with a role of *Mmp1* during cardiac aging.

## Discussion

### Conservation of cardiac aging processes

Age‐related remodeling causes mammalian hearts to become stiffer, more arrhythmic, and less contractile (Strait & Lakatta, 2012). However, the molecular mechanisms that cause these changes are not well understood. Fly hearts have significant genetic homology and proteomic similarity with mammalian hearts (Cammarato *et al*., 2011), and also show increased stiffness, arrhythmias and dysfunction with age (Ocorr *et al*., 2007a; Kaushik *et al*., 2012). Thus, *Drosophila* with its short lifespan and myriad of genetic tools can be a useful model organism to investigate the molecular causes of cardiac aging.

This study investigated the transcriptional changes that occur in aging fly hearts, and assessed whether these changes are conserved in aging rodent hearts. We found that genes involved in extra‐cellular matrix remodeling, mitochondrial metabolism, protein handling, and contractile function were altered in multiple aging fly and rodent heart datasets. However, no single process was found to be significantly enriched in aged genes among all data sets examined.

### Cardiac aging appears to be stochastic at the individual gene level

One of the most striking observations from this study is that surprisingly few specific genes were similarly altered across multiple aging cardiac transcriptomic datasets. This phenomenon has been previously ascribed to each experiment being conducted on animals of differing genetic backgrounds, with different microarray platforms, and in different laboratories. We propose that this result could also highlight the fact that each individual ‐ or at least individuals with similar genetic backgrounds – age through similar processes, but not identical transcriptional changes (Figs 1 and 2).

It has been well established that gene expression is highly variable between individual cells, and that the degree of variability is cell type specific (Dueck *et al*., 2015). Cell‐to‐cell transcriptional variability is significant in young mouse hearts and worsens with age (Bahar *et al*., 2006). For example, single cardiomyocytes from 27‐month‐old mice show much more variability in expression of housekeeper genes like *Gapdh*,* Actb*,* B2M* and *Tuba6*, and of cardiac specific genes like *Actc1* and *Myl2*, than cardiomyocytes from young (6‐month‐old) mice (Bahar *et al*., 2006). However, we saw similar transcriptional variability in both young and old fly hearts (Fig. S1, Supporting information).

Our single fly heart data and our meta‐analysis of published rodent cardiac aging data suggest that in aging cardiac tissue gene expression is highly stochastic. Differences in genetic aging would explain why the heritability of longevity in humans significantly varies by ethnicity and is estimated to be only 4–30% (Herskind *et al*., 1996; Lee *et al*., 2004). Moreover, a number of genome‐wide association studies (GWAS) on various human populations have uncovered very few genes that correlate with human longevity, and different sets of aging genes were resolved in each study (Murabito *et al*., 2012). Also, the AgeMAP study, which compared gene expression across different tissues in a number of species, found merely 22 genes to be similarly changed in the same tissues in mice and humans; only the electron transport chain gene set appeared altered in expression with age in human, mouse, and fly (Zahn *et al*., 2007). Our study used enrichment analyses with the most current ontologies to find many more biological processes that are likely to be conserved during aging (Fig. 2). All these data support the hypothesis that cardiac aging may occur by similar processes in different species, but is unlikely to be driven by changes in the exact same gene set in each individual.

### Developmental genes are important in cardiac aging

How can differing transcriptional changes during cardiac aging lead to a common functional decline at old age? Perhaps master regulators that control developmental processes also participate in aging; these molecules may activate different transcriptional targets in varying cellular milieus. We found that two such molecules, *Odd* and *miR‐1*, are potential contributors to cardiac aging in the *Drosophila* model.

The zinc‐finger transcription factor Odd is up‐regulated in old fly hearts (Fig. 4; Table S1, Supporting information). Odd was originally identified as a critical transcriptional regulator in patterning the body plan of the *Drosophila* embryo (Coulter & Wieschaus, 1988). Furthermore, its mammalian homolog, Odd‐skipped related 1 (Osr1), is required for heart and kidney development in mice (Wang *et al*., 2005). However, it was not known whether Odd contributes to aging‐related tissue remodeling. We found that cardiac‐specific knockdown of *Odd* decreases heart rate and increases arrhythmia in old flies – changes that are commonly seen in the hearts of elderly humans. The fact that knockdown of *Odd* exacerbates cardiac aging in the fly suggests that the increased *Odd* expression in aging hearts may be an attempt to compensate for functional decline. We found that cardiac‐specific over‐expression of *Odd* is detrimental in young flies, causing bradycardia, cardiac dilation and contractile defects, but has no effect on heart function in old flies. This also points to the possibility that the *Odd* up‐regulation, observed with age in old flies, may be a compensatory response to aging, and that *Odd* expression must be tightly regulated throughout life for optimal cardiac function. This is reminiscent of reports showing that dysregulation in cardiac expression of the KCNQ potassium channel, integrin‐linked kinase and β1‐integrin, or the transcription factor FoxO, leads to cardiac dysfunction and accelerated aging (Ocorr *et al*., 2007b; Nishimura *et al*., 2014; Blice‐Baum *et al*. 2017).

We found that several predicted Odd target genes are also up‐regulated with age (Fig. 4). These genes include the antimicrobial peptide‐encoding *LysX*. Cardiac‐specific *LysX* inhibition caused bradycardia (Fig. 5A), similar to that seen in Odd‐deficient hearts (Fig. 3A), and cardiac constriction (Fig. 5A), an opposite phenotype to that seen in Odd‐overexpressing hearts (Fig. 3B). These data support the hypothesis that *LysX* is an Odd target gene and that Odd may alter transcription of immune modulating genes as the heart ages.

We also discovered that *miR‐1* was down‐regulated with age and is predicted to target a number of the genes that were up‐regulated in the aged fly heart (Fig. 1). Like Odd, *miR‐1* is essential for heart development and function in both flies and mice (Kwon *et al*., 2005; Zhao *et al*., 2005; Qian *et al*., 2011). Furthermore, *miR‐1* expression decreases in human heart failure (Tritsch *et al*., 2013). We have previously shown that cardiac restricted *miR‐1* overexpression induces arrhythmias in young adult flies (Qian *et al*., 2011). Therefore, like Odd, *miR‐1* possibly regulates several of the age‐related gene changes in the fly heart.

Odd is a key developmental regulator. Our data support the intriguing hypothesis that developmental genes may also control aging, possibly acting through a different target gene set. This warrants further investigation of exactly which genes are targeted by Odd during embryonic development, adult maintenance, and age‐associated decline of the heart.

*Matrix metalloprotease 1* (*Mmp1*) was one of the genes we found to be up‐regulated with age and it is potentially targeted by both Odd and *miR‐1*. *Drosophila* Mmp1 has a number of mammalian Mmp homologues, including Mmp2, ‐9, ‐11, ‐14, and ‐15. Several of these are also altered in old rodent hearts (Table S3, Supporting information) and are known to contribute to cardiac pathology including contractile dysfunction (Spinale *et al*., 2009).

## Conclusion

This study shows that aging‐induced extra‐cellular matrix remodeling and changes in mitochondrial metabolism, protein handling, and contractile function are conserved in *Drosophila* hearts, mirroring several aging processes in mammalian hearts. We propose that diverse genetic mechanisms can lead to similar aging processes. While the processes themselves are evolutionarily conserved, there is little overlap of individual gene expression changes either between or within species. Therefore, we need to identify the types of regulatory mechanisms which drive or accompany these diverse aging events. Our data suggest that age‐dependent changes in heart physiology could be controlled, in part, by the transcription factor encoded by *Odd*.

## Materials and methods

### *Drosophila* stocks

*yw* and progeny of *yw* crossed to *GMH5‐Gal4* (Wessells *et al*., 2004) were used for microarray analysis of age‐related changes in the *Drosophila* heart. *Odd*‐TRiP RNAi (stock number 34328) and *Odd*‐OE lines were obtained from Bloomington Stock Center (stock number 9902). The *Mmp1‐*OE line (genotype: *w*; {*UAS‐Mmp1.f1, w*+}/*TM3*), stock number AMP1037) was obtained from Andrea Page McCaw. LysX‐RNAi line (stock number v49896) was obtained from VDRC. Cardiac driver lines *Hand4.2‐Gal4 and Hand‐GS‐Gal4* were as used previously in Kaushik *et al*. (2012), Nishimura *et al*. (2014) and Monnier *et al*. (2012).

### Aging flies

All flies were collected within 24 h of eclosing and aged in vials of standard fly media at 25 °C with 12 h light‐dark cycles. Flies were transferred into fresh food vials twice a week. Only female flies were collected and used for all experiments.

### RNA isolation and cDNA production, labeling and fragmentation

*Drosophila* hearts from 1‐ and 5‐week‐old *yw* wildtype or *yw x GMH5* flies (*n* = 30) were isolated (Fink *et al*., 2009), removed and homogenized in TRIzol (Invitrogen Life Sciences). Total RNA was precipitated and purified by miRNeasy mini‐column (Qiagen) according to the manufacturer's instructions. The recovered RNA was subjected to first and second strand cDNA synthesis, amplification, and purification using the WT‐ovation Pico RNA Amplification System (NuGEN), Agencourt RNAClean purification beads (Beckman Coulter), and DNA Clean and Concentrator‐25s columns (Zymo Research). cDNA was fragmented and labeled using the FL‐ovation cDNA Biotin Module V2 (NuGEN).

### Microarray analysis

Hybridization of biotin labeled cardiac cDNA from each fly group to Affymetrix GeneChip arrays, washing, staining, and scanning were carried out by the UCSD/Veterans Medical Research Foundation GeneChip Microarray Core facility using standard protocols. Five biological replicates (for each age and genotype group) were performed. The cel files were quality controlled with the R package AffyQCReport (Parman *et al*., 2013) and resulted in the following numbers of replicates per group: 4 of the *yw* × *GMH5* 1 week, 3 of the *yw* × *GMH5* 5 week, 3 of the *yw* 1 week and 3 of the *yw* 5 week. The limma package (Smyth & Speed, 2003) was then used to determine genotype, aging, and interaction *P* values.

### Gene ontology and microRNA binding site enrichment analysis

GO‐Elite (Zambon *et al*., 2012) was used to identify Gene Ontology (GO) and miR binding site enrichment in up‐ and down‐regulated aging genes. Ensembl‐miR associations were obtained from the program GO‐Elite (Emig *et al*., 2010) which incorporates miR binding site predictions from TargetScan, RNAhybrid, miRanda, miRBase, and Pictar databases. To provide a more conservative set of predictions for human, mouse, and rat, only miRs found in more than one database were included. Details on this process are available from http://www.altanalyze.org/help.htm#microrna. Associated EntrezGene relationships were obtained using the Ensembl‐EntrezGene relationship files in GO‐Elite.

### Meta‐analysis

11 rodent aging heart datasets were compared to two aging fly heart datasets using the following analyses. All of the datasets with the exception of the fly dataset from Monnier *et al*. (2012) were generated with Affymetrix 3′ expression microarrays. All raw Affymetrix.cel files were downloaded and submitted for quality control (QC) analysis using the Affymetrix QC report (Parman *et al*., 2013). Samples failing QC standards were removed prior to normalization. Each individual dataset, with the exception of the non‐affymetrix data from Monnier, was then background subtracted and normalized with RMA in R (Irizarry *et al*., 2003). Each experiment's internal control data (the “young” samples) were used to compute fold changes and *P* values against the aged groups. Identical fold (|fold|> 1.2) and *P* value (*P* < 0.05) cutoffs were used to define age responsive transcripts. GO term enrichment was computed with GO‐Elite. We then filtered all significantly enriched GO categories (*Z* > 2) in our *yw* × *GMH5* aging genes for GO‐terms also significantly enriched in at least four or more of the other datasets. These pathways were then clustered by *Z* score using complete linkage clustering. GO‐terms for datasets that fell below the significance threshold of (*Z* > 2) were reset to a *Z* score of 0 prior to clustering to facilitate heat map visualization.

### Identification of evolutionarily conserved Odd consensus elements

oPOSSUM single site analysis (Ho Sui *et al*., 2007) was used to analyze promoter sequences for enrichment of evolutionarily conserved transcription factor binding site sequence motifs. Occurrence frequencies of transcription factor binding sites, based on insect and vertebrate position weight matrices from the JASPAR database, were computed within 2 kb of the transcription start site of the ~237 upregulated aging genes (aging *P*‐value < 0.005, interaction *P*‐value > 0.05) using the recommended default query parameters recommended by the website.

### Nanofluidic qPCR of single fly hearts

Individual hearts from female 1‐ and 5‐week‐old flies were isolated and snap frozen in 8 μL of water. Each heart was then brought up to a final volume of 40 μL of lysis buffer (LB) (0.25% NP40 in water). Lysis was carried out by heating individual hearts in LB for 2 min at 98 °C. The lysed hearts were then briefly centrifuged, and 30 μL transferred to a fresh tube. Reverse transcription was carried out by using 3.3 μL of lysed heart (~8% of a single heart) in a 5 μL final reaction volume using the VILO Reaction mix as per the manufacturer's instructions (Life Technologies), resulting in a final volume of 6 μL of RT cDNA per heart. For use with the BioMark platform, we then carried out pre‐amplification for 48 genes using a Fluidigm Master Mix (Fluidigm corp) (3 μL), 10× single target amplification (STA) (1.5 μL of 10× STA – 100 μm primers), 0.5 m EDTA (pH 8) (0.075 μL), and water, to a final volume of 9 μL. Pre‐amplification was then carried out in a volume of 15 μL (pre‐amp cycling – 95 °C for 2 min, and 20 cycles of 96 °C 5 s, 60 °C for 4 min). For removal of single stranded DNA prior to nanofluidic cycling, 6 μL of Exosap solution (4.2 μL of water, 0.6 μL of Exonuclease 1 Rn Buffer, Exonuclease 1 (20 units μL^−1^, New England Biolabs) was added to the 15 μL final reaction volume of the RT step. The resulting 21 μL final volume was then incubated at 37 °C for 30 min and then heat inactivated at 80 °C for 15 min. The pre‐amplified volume (21 μL) was then diluted 10‐fold in DNA suspension buffer (Teknova), and stored at −20 °C prior to running on the chip. Pre‐amplified products for each individual fly heart were then assayed using Fluidigm's 48.48 nanofluidic qPCR arrays on a Biomark system (Fluidigm), according to their protocols. Biotium's EvaGreen DNA binding dye was used to detect amplified products according to Fluidigm's protocols.

### RNA *in situ* hybridization

RNA *in situ* hybridization was performed as in Viswanathan *et al*. (2016) and Blice‐Baum AC *et al*. (in press, *Aging Cell*) with modifications: probes against *Odd‐skipped* and *Gapdh1* were generated using the RNAscope (ACD) platform, and hybridized following the manufacturer's protocol. Labeled specimens were immediately mounted in Prolong Gold (Molecular Probes), and imaged the following day on an Imager.Z1 equipped with an OrcaFlash 4LT camera (Hamamatsu) and Apotome.2 (Zeiss), using ZEN software (2.3). 16‐bit dual channel images were analyzed using Fiji/ImageJ. Particles, representing individual messages, were quantified.

### microRNA isolation and quantification

Total RNA was extracted from 20‐30 female 1 week and 5 week hearts using the miRNeasy kit. RT‐PCR was done using Taqman microRNA assays according to the manufacturer's protocol (Life Technology, Applied Byosystem, Carlsbad, CA, USA). A total of nine PCR experiments from triplicate biological samples were analyzed and normalized with *2S*.

### *In vivo* validation

Genes of interest were knocked down or over expressed specifically in the heart using Hand4.2‐Gal4 in conjunction with UAS‐RNAi lines (VDRC KK library, Bloomington TRiP library) or UAS‐over expressing lines. *In vivo* cardiac parameters were assessed in female flies at different ages using the semi‐intact preparation outlined in (Fink *et al*., 2009).

### Statistics

For cluster analysis of aging genes the following LIMMA‐derived statistical filters were used: Aging *P*‐value < 0.005, interaction *P*‐value > 0.05. This resulted in 260 up‐regulated and 202 down‐regulated aging probe sets. For the cross species meta‐analysis of functional groups, a less stringent filter was used: Aging *P*‐value < 0.05, interaction *P*‐value > 0.05. This resulted in 720 up‐regulated and 763 down‐regulated aging probe sets. The significance of the *in vivo* studies of cardiac function was assessed via analysis of variance (ANOVA), and Tukey's multiple comparison using GraphPad Prism^®^. Values are presented as means ± SEM. *P* values < 0.05 were considered significant. For statistical analysis of the nanofluidic data, all statistical comparisons between age‐groups were tested using a non‐parametric randomization test using r statistical software under the DAAG software package. Treatment groups were compared and the null was rejected if *P* < 0.05. This statistical test was employed to avoid employing an assumption of a normal distribution of sample values in the targeted hearts from different ages.

## Authors' contributions

LC, AC, AZ, SIB, SM, and RB designed experiments and wrote the manuscript. AZ and ZZ performed the bioinformatics analyses. LC, AC, AZ, ZZ, ET, GV, MM, JC, and SM performed experiments and statistical analysis. AC, SIB, and RB provided funding. All authors read and approved the final manuscript.

## Funding

This work was funded by the following grants: American Heart Association grant 10SDG4180089 (to AC); AFAR Research Grant (to AC); CSUPERB (CSU Program for Education and Research in Biotechnology) Grant (to AC and SIB); American Heart Association grant 10SDG2630130 (to AZ); NIH R01 GM32443 (to SIB); Larry L. Hillblom Foundation 2009‐A‐001‐CTR (to SM); NIH P01 AG033456; NIH P01 HL098053, NIH R01 HL54732 and a Senior Scholar Award from the Ellison Medical Foundation (to RB).

## Conflict of interest

None declared.

## Supporting information


**Fig. S1** Heart‐to‐heart variability in normalized gene expression.

**Fig. S2** Induced *Odd* expression in old flies.

**Fig. S3** miR‐1 expression.

**Table S1** Age‐related genes.

**Table S2** GO terms enriched in up‐ and down‐regulated genes.

**Table S3** Genes differentially regulated in fly and rodent gene sets.

**Table S4** oPOSSUM transcription factor binding site enrichment analysis in proximal promoters of aging responsive genes.
