# Supplementary figures and images for: Expression patterns of cardiac aging in Drosophila
Source: Aging Cell. 2017 Jan 16;16(1):82–92. doi: 10.1111/acel.12559 (PMC5242310; doi:10.1111/acel.12559)

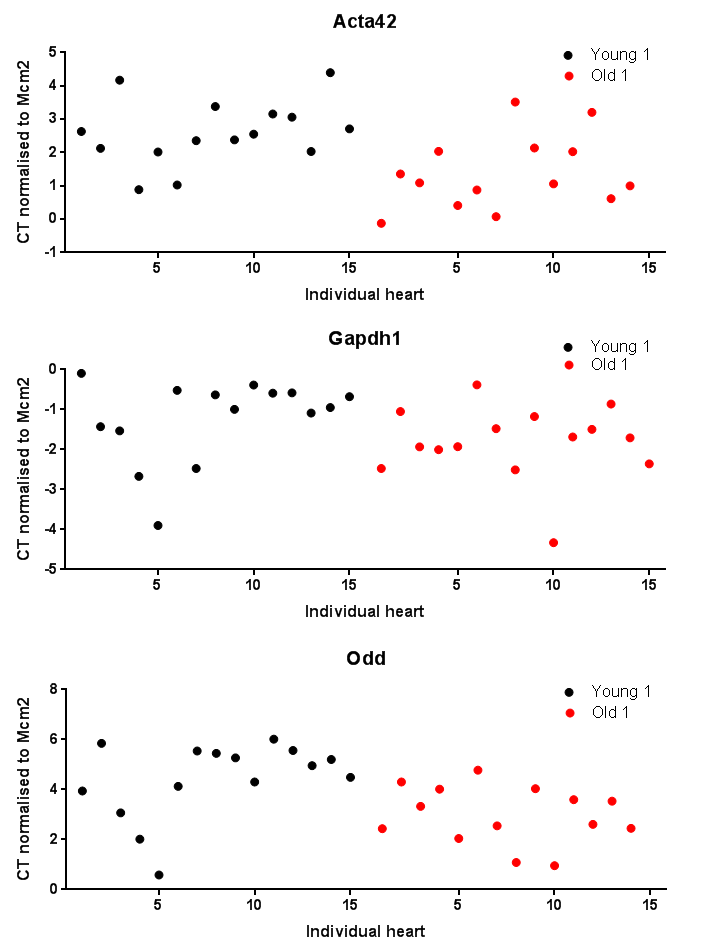

Supplement: Supplementary file 1 — Fig. S1 Heart‐to‐heart variability in normalized gene expression. [file ACEL-16-82-s001.tif]

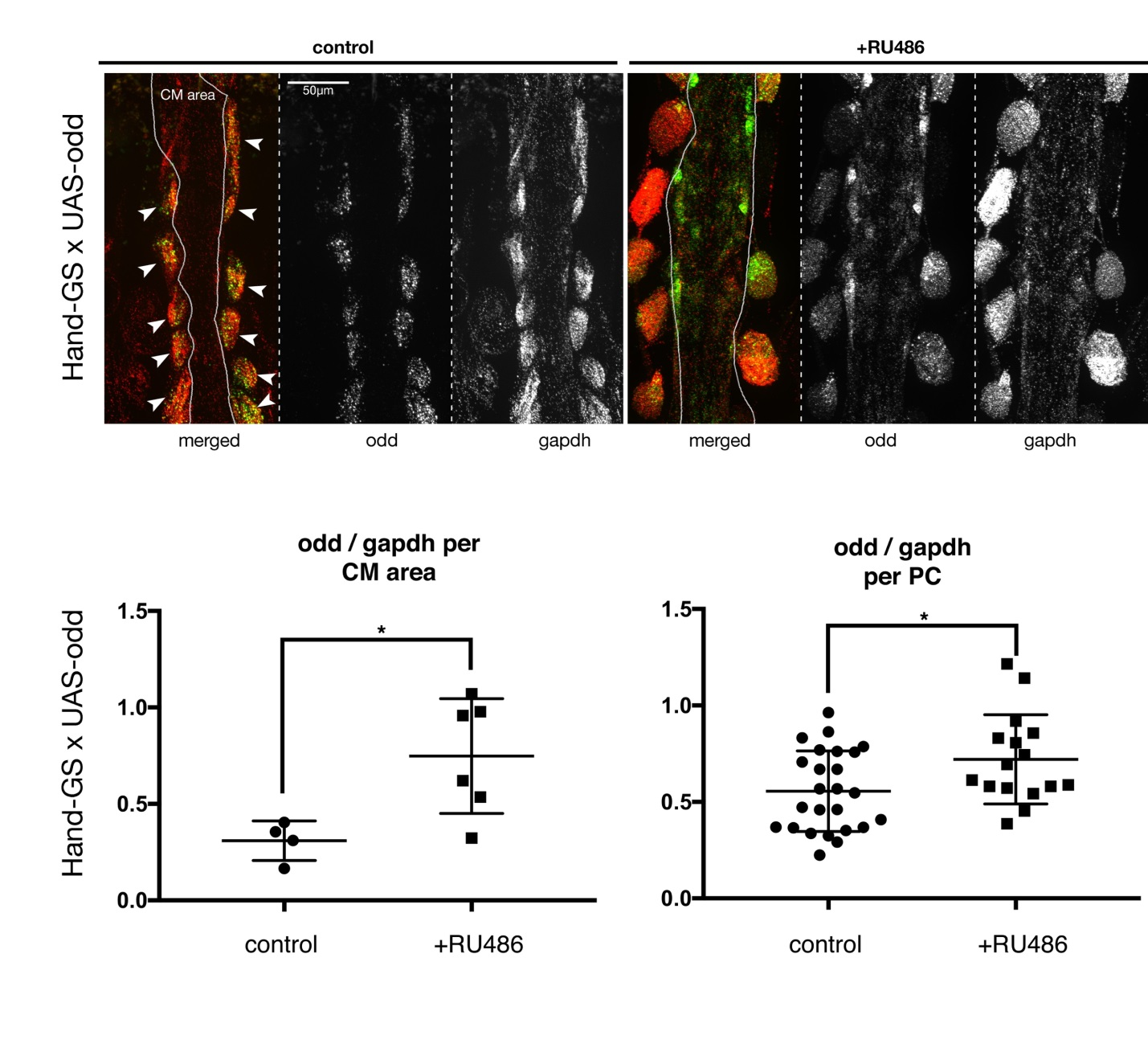

Supplement: Supplementary file 2 — Fig. S2 Induced Odd expression in old flies. [file ACEL-16-82-s002.tif]

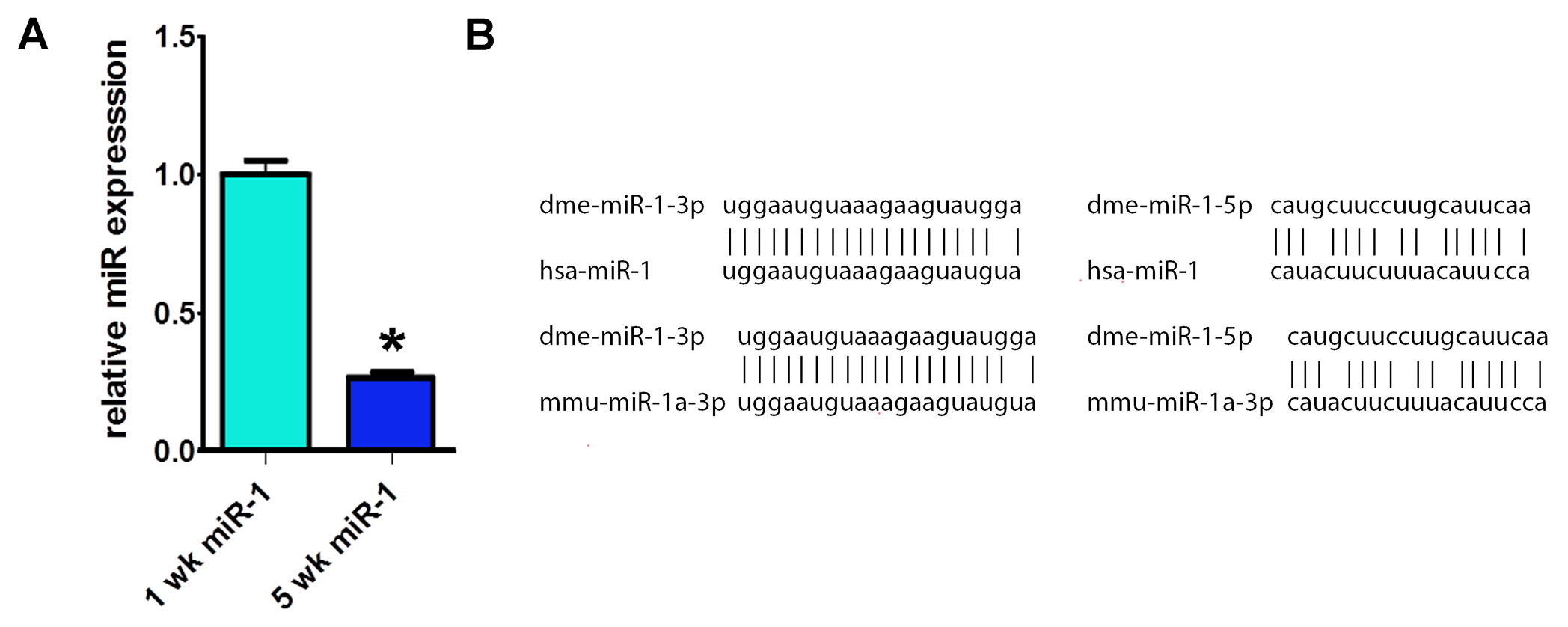

Supplement: Supplementary file 3 — Fig. S3 miR‐1 expression. [file ACEL-16-82-s003.tif]
