# Supplementary material for: Expression patterns of cardiac aging in Drosophila
Source: Aging Cell. 2017 Jan 16;16(1):82–92. doi: 10.1111/acel.12559 (PMC5242310; doi:10.1111/acel.12559)
Supplement: Supplementary file 5 — Table S2 GO terms enriched in up‐ and down‐regulated genes. [file ACEL-16-82-s005.pdf]

Supplemental Table 2. GO terms enriched in up- and down-regulated genes

UP-REGULATED

Ontology-ID	Ontology Name	Ontology Type	No. Changed	No. Measured	No. in Ontolog	% Changed	% Present	Z Score	Permute P	Adjusted P
GO:0030554	adenyl nucleotide binding	molecular_fun	25	850	867	2.94	98.04	2.05083803	0.0595	1
GO:0032559	adenyl ribonucleotide binding	molecular_fun	25	849	866	2.94	98.04	2.05704183	0.05975	1
GO:0004812	aminoacyl-tRNA ligase activity	molecular_fun	4	68	69	5.88	98.55	2.29850905	0.01875	1
GO:0005524	ATP binding	molecular_fun	25	847	864	2.95	98.03	2.06947206	0.062	1
GO:0043190	ATP-binding cassette (ABC) transpor	cellular_comp	3	43	43	6.98	100.00	2.33981471	0.0355	1
GO:0022403	cell cycle phase	biological_pro	13	222	222	5.86	100.00	4.15391262	0	0.32425
GO:0022402	cell cycle process	biological_pro	19	522	524	3.64	99.62	2.75117252	0.0025	0.60796875
GO:0000267	cell fraction	cellular_comp	7	118	118	5.93	100.00	3.07366599	0.0015	0.486375
GO:0044464	cell part	cellular_comp	136	6053	6530	2.25	92.70	2.07343074	0.82125	1
GO:0071842	cellular component organization at c	biological_pro	37	1349	1468	2.74	91.89	2.09309422	0.075	1
GO:0044255	cellular lipid metabolic process	biological_pro	10	197	198	5.08	99.49	3.11902121	0.00125	0.46321429
GO:0044427	chromosomal part	cellular_comp	11	267	377	4.12	70.82	2.51192043	0.0025	0.60796875
GO:0051276	chromosome organization	biological_pro	11	297	406	3.70	73.15	2.13318484	0.014	1
GO:0000781	chromosome, telomeric region	cellular_comp	3	11	11	27.27	100.00	5.99694847	0.00075	0.3891
GO:0008234	cysteine-type peptidase activity	molecular_fun	4	77	78	5.19	98.72	2.01402121	0.03425	1
GO:0015036	disulfide oxidoreductase activity	molecular_fun	3	29	29	10.34	100.00	3.21881951	0.007	1
GO:0006259	DNA metabolic process	biological_pro	10	250	251	4.00	99.60	2.29168345	0.0095	1
GO:0008094	DNA-dependent ATPase activity	molecular_fun	3	37	37	8.11	100.00	2.66271272	0.02025	1
GO:0004519	endonuclease activity	molecular_fun	3	49	50	6.12	98.00	2.06989569	0.041	1
GO:0005789	endoplasmic reticulum membrane	cellular_comp	7	145	145	4.83	100.00	2.45411219	0.00575	1
GO:0031012	extracellular matrix	cellular_comp	4	51	51	7.84	100.00	2.99251915	0.01375	1
GO:0044421	extracellular region part	cellular_comp	9	166	178	5.42	93.26	3.1797714	0.00125	0.46321429
GO:0030718	germ-line stem cell maintenance	biological_pro	3	36	36	8.33	100.00	2.72314119	0.018	1
GO:0046486	glycerolipid metabolic process	biological_pro	4	60	61	6.67	98.36	2.59384088	0.02025	1
GO:0006650	glycerophospholipid metabolic proce	biological_pro	4	53	54	7.55	98.15	2.89648329	0.011	1
GO:0006505	GPI anchor metabolic process	biological_pro	3	25	25	12.00	100.00	3.58043554	0.0065	1
GO:0004553	hydrolase activity, hydrolyzing O-gly	molecular_fun	5	103	108	4.85	95.37	2.08393576	0.019	1
GO:0015103	inorganic anion transmembrane tran	molecular_fun	3	44	44	6.82	100.00	2.29162236	0.03475	1
GO:0005626	insoluble fraction	cellular_comp	6	113	113	5.31	100.00	2.53162386	0.00575	1
GO:0008299	isoprenoid biosynthetic process	biological_pro	3	15	15	20.00	100.00	4.98892955	0.001	0.40957895
GO:0006720	isoprenoid metabolic process	biological_pro	3	22	22	13.64	100.00	3.90760055	0.0025	0.60796875
GO:0000776	kinetochore	cellular_comp	5	29	29	17.24	100.00	5.87767728	0	0.32425
GO:0016876	ligase activity, forming aminoacyl-tR	molecular_fun	4	68	69	5.88	98.55	2.29850905	0.01875	1
GO:0016875	ligase activity, forming carbon-oxyge	molecular_fun	4	68	69	5.88	98.55	2.29850905	0.01875	1
GO:0009057	macromolecule catabolic process	biological_pro	8	199	202	4.02	98.51	2.060271	0.01225	1
GO:0005624	membrane fraction	cellular_comp	6	109	109	5.50	100.00	2.63213043	0.00475	0.97275
GO:0004222	metalloendopeptidase activity	molecular_fun	5	82	82	6.10	100.00	2.66551503	0.011	1
GO:0007552	metamorphosis	biological_pro	3	27	28	11.11	96.43	3.39059555	0.0105	1
GO:0005792	microsome	cellular_comp	5	88	88	5.68	100.00	2.48210813	0.00875	1
GO:0031023	microtubule organizing center organ	biological_pro	3	48	48	6.25	100.00	2.11189276	0.04325	1
GO:0007067	mitosis	biological_pro	8	138	138	5.80	100.00	3.2128063	0.002	0.53668966
GO:0007094	mitotic cell cycle spindle assembly cl	biological_pro	3	12	12	25.00	100.00	5.70068451	0.00025	0.32425
GO:0071174	mitotic cell cycle spindle checkpoint	biological_pro	3	12	12	25.00	100.00	5.70068451	0.00025	0.32425
GO:0004497	monooxygenase activity	molecular_fun	6	116	117	5.17	99.15	2.45905722	0.01	1
GO:0045786	negative regulation of cell cycle	biological_pro	3	39	39	7.69	100.00	2.54799354	0.02425	1
GO:0010948	negative regulation of cell cycle proc	biological_pro	3	29	29	10.34	100.00	3.21881951	0.0085	1
GO:0045839	negative regulation of mitosis	biological_pro	3	16	16	18.75	100.00	4.79502808	0.001	0.40957895
GO:0045841	negative regulation of mitotic metap	biological_pro	3	14	14	21.43	100.00	5.20196131	0.00075	0.3891
GO:0051784	negative regulation of nuclear divisio	biological_pro	3	16	16	18.75	100.00	4.79502808	0.001	0.40957895
GO:0010639	negative regulation of organelle orga	biological_pro	3	40	40	7.50	100.00	2.49345246	0.02075	1
GO:0000280	nuclear division	biological_pro	8	141	141	5.67	100.00	3.14259575	0.002	0.53668966
GO:0048285	organelle fission	biological_pro	8	146	146	5.48	100.00	3.02957936	0.00275	0.62942647
GO:0006996	organelle organization	biological_pro	31	974	1091	3.18	89.28	2.77321697	0.00575	1
GO:0016667	oxidoreductase activity, acting on a :	molecular_fun	3	41	41	7.32	100.00	2.44064116	0.01825	1
GO:0046488	phosphatidylinositol metabolic proce	biological_pro	4	44	45	9.09	97.78	3.37164511	0.0075	1
GO:0046148	pigment biosynthetic process	biological_pro	4	51	51	7.84	100.00	2.99251915	0.0065	1
GO:0042440	pigment metabolic process	biological_pro	4	69	69	5.80	100.00	2.26466333	0.024	1
GO:0006779	porphyrin biosynthetic process	biological_pro	3	11	11	27.27	100.00	5.99694847	0.0015	0.486375
GO:0006778	porphyrin metabolic process	biological_pro	3	12	12	25.00	100.00	5.70068451	0.0015	0.486375
GO:0007088	regulation of mitosis	biological_pro	4	46	46	8.70	100.00	3.25566561	0.00725	1
GO:0030071	regulation of mitotic metaphase/ana	biological_pro	4	24	24	16.67	100.00	5.14429492	0.001	0.40957895
GO:0051783	regulation of nuclear division	biological_pro	4	46	46	8.70	100.00	3.25566561	0.00725	1
GO:0005819	spindle	cellular_comp	4	43	43	9.30	100.00	3.43227416	0.00375	0.83378571
GO:0071173	spindle assembly checkpoint	biological_pro	3	12	12	25.00	100.00	5.70068451	0.00025	0.32425
GO:0031577	spindle checkpoint	biological_pro	3	12	12	25.00	100.00	5.70068451	0.00025	0.32425
GO:0019827	stem cell maintenance	biological_pro	3	47	47	6.38	100.00	2.15500381	0.0425	1
GO:0044456	synapse part	cellular_comp	6	125	126	4.80	99.21	2.25432652	0.04325	1
GO:0033014	tetrapyrrole biosynthetic process	biological_pro	3	13	13	23.08	100.00	5.43768722	0.00175	0.50438889
GO:0033013	tetrapyrrole metabolic process	biological_pro	3	14	14	21.43	100.00	5.20196131	0.00175	0.50438889
GO:0000151	ubiquitin ligase complex	cellular_comp	3	48	48	6.25	100.00	2.11189276	0.037	1
GO:0042598	vesicular fraction	cellular_comp	5	88	88	5.68	100.00	2.48210813	0.00875	1

DOWN-REGULATED

Ontology-ID	Ontology Name	Ontology Type	Number Chang	Number Meas	Number in Ont	Percent Chang	Percent Preser	Z Score	PermuteP	AdjustedP
GO:0022804	active transmembrane transporter a	molecular_fun	12	372	377	3.23	98.67	2.85693886	0.003	1
GO:0046164	alcohol catabolic process	biological_pro	3	51	51	5.88	100.00	2.6211722	0.03525	1
GO:0005275	amine transmembrane transporter a	molecular_fun	4	59	59	6.78	100.00	3.39410121	0.007	1
GO:0015837	amine transport	biological_pro	3	44	44	6.82	100.00	2.95032707	0.02025	1
GO:0015171	amino acid transmembrane transpor	molecular_fun	4	54	54	7.41	100.00	3.63031984	0.0045	1
GO:0006865	amino acid transport	biological_pro	3	40	40	7.50	100.00	3.17121036	0.01375	1
GO:0006022	aminoglycan metabolic process	biological_pro	5	140	150	3.57	93.33	2.07566587	0.0175	1
GO:0006820	anion transport	biological_pro	3	53	54	5.66	98.15	2.53781528	0.024	1
GO:0043492	ATPase activity, coupled to movemer	molecular_fun	6	147	151	4.08	97.35	2.64466965	0.00875	1
GO:0042626	ATPase activity, coupled to transmer	molecular_fun	6	147	151	4.08	97.35	2.64466965	0.00875	1
GO:0043190	ATP-binding cassette (ABC) transpor	cellular_comp	3	43	43	6.98	100.00	3.00297625	0.01475	1
GO:0005509	calcium ion binding	molecular_fun	8	231	236	3.46	97.88	2.53938397	0.01075	1
GO:0016052	carbohydrate catabolic process	biological_pro	5	85	86	5.88	98.84	3.38915012	0.0025	1
GO:0005975	carbohydrate metabolic process	biological_pro	13	408	422	3.19	96.68	2.92947458	0.002	1
GO:0046943	carboxylic acid transmembrane trans	molecular_fun	7	86	87	8.14	98.85	5.15399415	0.00025	0.3891
GO:0046942	carboxylic acid transport	biological_pro	5	54	55	9.26	98.18	4.7629919	0.00025	0.3891
GO:0044275	cellular carbohydrate catabolic proce	biological_pro	4	51	51	7.84	100.00	3.78652351	0.00275	1
GO:0044262	cellular carbohydrate metabolic proc	biological_pro	6	184	188	3.26	97.87	2.03167306	0.032	1
GO:0008061	chitin binding	molecular_fun	4	99	107	4.04	92.52	2.13138702	0.02625	1
GO:0006030	chitin metabolic process	biological_pro	5	113	121	4.42	93.39	2.61956867	0.0085	1
GO:0048512	circadian behavior	biological_pro	4	45	45	8.89	100.00	4.13950999	0.0075	1
GO:0007623	circadian rhythm	biological_pro	4	55	55	7.27	100.00	3.58080681	0.01375	1
GO:0022410	circadian sleep/wake cycle process	biological_pro	3	17	17	17.65	100.00	5.54168909	0.00425	1
GO:0046843	dorsal appendage formation	biological_pro	3	34	34	8.82	100.00	3.56468821	0.01275	1
GO:0006091	generation of precursor metabolites	biological_pro	6	136	149	4.41	91.28	2.86416361	0.005	1
GO:0006007	glucose catabolic process	biological_pro	3	43	43	6.98	100.00	3.00297625	0.01425	1
GO:0006006	glucose metabolic process	biological_pro	3	53	53	5.66	100.00	2.53781528	0.02875	1
GO:0015020	glucuronosyltransferase activity	molecular_fun	3	34	34	8.82	100.00	3.56468821	0.0055	1
GO:0006096	glycolysis	biological_pro	3	36	36	8.33	100.00	3.42375988	0.00775	1
GO:0019320	hexose catabolic process	biological_pro	3	43	43	6.98	100.00	3.00297625	0.01425	1
GO:0016820	hydrolase activity, acting on acid anl	molecular_fun	6	158	162	3.80	97.53	2.44450029	0.0135	1
GO:0004553	hydrolase activity, hydrolyzing O-gly	molecular_fun	4	103	108	3.88	95.37	2.04157342	0.03025	1
GO:0008289	lipid binding	molecular_fun	5	138	148	3.62	93.24	2.11140157	0.04075	1
GO:0005811	lipid particle	cellular_comp	9	251	290	3.59	86.55	2.81241724	0.00575	1
GO:0007626	locomotory behavior	biological_pro	4	103	103	3.88	100.00	2.04157342	0.09275	1
GO:0016020	membrane	cellular_comp	36	1789	1825	2.01	98.03	2.07206383	0.06075	1
GO:0046365	monosaccharide catabolic process	biological_pro	3	44	44	6.82	100.00	2.95032707	0.0155	1
GO:0008045	motor axon guidance	biological_pro	3	37	38	8.11	97.37	3.35720155	0.018	1
GO:0051129	negative regulation of cellular compo	biological_pro	3	68	68	4.41	100.00	2.01900274	0.06075	1
GO:0007528	neuromuscular junction developmen	biological_pro	3	43	43	6.98	100.00	3.00297625	0.0425	1
GO:0007218	neuropeptide signaling pathway	biological_pro	3	36	37	8.33	97.30	3.42375988	0.006	1
GO:0071705	nitrogen compound transport	biological_pro	3	53	53	5.66	100.00	2.53781528	0.035	1
GO:0005342	organic acid transmembrane transpo	molecular_fun	7	88	89	7.95	98.88	5.06939049	0.00025	0.3891
GO:0015849	organic acid transport	biological_pro	5	54	55	9.26	98.18	4.7629919	0.00025	0.3891
GO:0071702	organic substance transport	biological_pro	7	125	126	5.60	99.21	3.85384544	0.0005	0.6485
GO:0005976	polysaccharide metabolic process	biological_pro	7	153	163	4.58	93.87	3.20925573	0.0015	1
GO:0006813	potassium ion transport	biological_pro	4	45	46	8.89	97.83	4.13950999	0.0065	1
GO:0015405	P-P-bond-hydrolysis-driven transme	molecular_fun	6	168	172	3.57	97.67	2.27669528	0.0185	1
GO:0015399	primary active transmembrane trans	molecular_fun	6	168	172	3.57	97.67	2.27669528	0.0185	1
GO:0050795	regulation of behavior	biological_pro	3	26	26	11.54	100.00	4.26688931	0.01025	1
GO:0042752	regulation of circadian rhythm	biological_pro	4	34	34	11.76	100.00	4.99084684	0.00225	1
GO:0042749	regulation of circadian sleep/wake c	biological_pro	3	15	15	20.00	100.00	5.96220478	0.003	1
GO:0031644	regulation of neurological system pr	biological_pro	4	36	36	11.11	100.00	4.80986252	0.00475	1
GO:0050804	regulation of synaptic transmission	biological_pro	4	36	36	11.11	100.00	4.80986252	0.00475	1
GO:0044057	regulation of system process	biological_pro	4	58	58	6.90	100.00	3.43917146	0.015	1
GO:0051969	regulation of transmission of nerve i	biological_pro	4	36	36	11.11	100.00	4.80986252	0.00475	1
GO:0035159	regulation of tube length, open trach	biological_pro	3	24	24	12.50	100.00	4.49066975	0.0055	1
GO:0009605	response to external stimulus	biological_pro	9	318	322	2.83	98.76	2.04044508	0.04925	1
GO:0007622	rhythmic behavior	biological_pro	4	48	48	8.33	100.00	3.95556831	0.01025	1
GO:0048511	rhythmic process	biological_pro	4	56	56	7.14	100.00	3.53247782	0.01475	1
GO:0007608	sensory perception of smell	biological_pro	4	95	96	4.21	98.96	2.22577612	0.036	1
GO:0015081	sodium ion transmembrane transpor	molecular_fun	4	85	88	4.71	96.59	2.48507738	0.02	1
GO:0005214	structural constituent of chitin-basec	molecular_fun	5	105	112	4.76	93.75	2.8124184	0.00725	1
GO:0042302	structural constituent of cuticle	molecular_fun	8	115	122	6.96	94.26	4.90896918	0.00025	0.3891
GO:0005198	structural molecule activity	molecular_fun	13	473	485	2.75	97.53	2.35572597	0.00825	1
GO:0007416	synapse assembly	biological_pro	3	34	34	8.82	100.00	3.56468821	0.018	1
GO:0050808	synapse organization	biological_pro	3	66	66	4.55	100.00	2.0793591	0.1065	1
GO:0051124	synaptic growth at neuromuscular ju	biological_pro	3	17	17	17.65	100.00	5.54168909	0.002	1
GO:0003008	system process	biological_pro	11	411	417	2.68	98.56	2.06598143	0.04775	1
GO:0016757	transferase activity, transferring glyc	molecular_fun	6	172	175	3.49	98.29	2.21290776	0.0135	1
GO:0016758	transferase activity, transferring hex	molecular_fun	6	141	144	4.26	97.92	2.76176834	0.0055	1
GO:0022857	transmembrane transporter activity	molecular_fun	20	744	760	2.69	97.89	2.85172053	0.002	1
GO:0022832	voltage-gated channel activity	molecular_fun	3	53	53	5.66	100.00	2.53781528	0.036	1
GO:0005244	voltage-gated ion channel activity	molecular_fun	3	53	53	5.66	100.00	2.53781528	0.036	1
