# Supplementary material for: Expression patterns of cardiac aging in Drosophila
Source: Aging Cell. 2017 Jan 16;16(1):82–92. doi: 10.1111/acel.12559 (PMC5242310; doi:10.1111/acel.12559)
Supplement: Supplementary file 7 — Table S4 oPOSSUM transcription factor binding site enrichment analysis in proximal promoters of aging responsive genes. [file ACEL-16-82-s007.pdf]

Supplemental Table-4. Filtered (Z≥4) oPPOSSUM enrichemnt analysis of transcription factor binding sites within 2kb of the transcriptional start site of Up and Down-regulated Aging transcripts (Aging P<0.005, Interaction P>0.05)

Group	TF	Class	Family	Target gene hits	Target gene non-hits	Background gene hits	Background gene non-hits	Target TFBS hits	Target TFBS nucleotide rate	Background TFBS hits	Background TFBS nucleotide rate	Z-score
Genes Upregulated Age	odd	Zinc-coordinating	BetaBetaAlpha-zinc finger	51	186	3142	9735	81	0.00452	4581	0.0033	9.4
Genes Upregulated Age	Esrrb	Zinc-coordinating	Hormone-nuclear Receptor	71	166	4804	8073	143	0.0087	8841	0.00694	9.4
Genes Upregulated Age	Sox2	Other Alpha-Helix	High Mobility Group	27	210	1717	11160	39	0.00297	2060	0.00202	9.3
Genes Upregulated Age	Optix	Helix-Turn-Helix	Homeo	149	88	9092	3785	729	0.0185	49496	0.0162	8.0
Upregulated Aging Genes	ovo	Zinc-coordinating	BetaBetaAlpha-zinc finger	117	120	7707	5170	384	0.0175	26065	0.0154	7.8
Upregulated Aging Genes	prd	Helix-Turn-Helix	Homeo	117	120	7707	5170	384	0.0175	26065	0.0154	7.8
Upregulated Aging Genes	Pou5f1	Helix-Turn-Helix	Homeo	39	198	2631	10246	59	0.00449	3527	0.00346	7.7
Upregulated Aging Genes	Myc	Zipper-Type	Helix-Loop-Helix	69	168	4876	8001	141	0.00715	8925	0.00584	7.6
Upregulated Aging Genes	RORA_1	Zinc-coordinating	Hormone-nuclear Receptor	71	166	4500	8377	124	0.00629	7831	0.00513	7.2
Upregulated Aging Genes	ESR2	Zinc-coordinating	Hormone-nuclear Receptor	8	229	359	12518	8	0.00073	368	0.000434	6.3
Upregulated Aging Genes	D	Other Alpha-Helix	High Mobility Group	116	121	7050	5827	302	0.0168	21057	0.0152	6.1
Upregulated Aging Genes	ESR1	Zinc-coordinating	Hormone-nuclear Receptor	1	236	23	12854	1	0.000101	23	3.01E-05	5.6
Upregulated Aging Genes	tin	Helix-Turn-Helix	Homeo	106	131	7112	5765	320	0.013	22332	0.0117	5.3
Upregulated Aging Genes	kni	Zinc-coordinating	Hormone-nuclear Receptor	53	184	3500	9377	79	0.00481	5231	0.00411	4.8
Upregulated Aging Genes	so	Helix-Turn-Helix	Homeo	131	106	8302	4575	504	0.0153	35795	0.0141	4.8
Upregulated Aging Genes	RXR::RAR_DR	Zinc-coordinating	Hormone-nuclear Receptor	6	231	338	12539	7	0.000603	354	0.000394	4.6
Upregulated Aging Genes	NFYA	Other Alpha-Helix	NFY CCAAT-binding	52	185	3325	9552	71	0.00576	4793	0.00502	4.6
Upregulated Aging Genes	FOXF2	Winged Helix-Turn-Heli	Forkhead	70	167	4241	8636	103	0.00731	7073	0.00648	4.6
Upregulated Aging Genes	Myf	Zipper-Type	Helix-Loop-Helix	81	156	5224	7653	153	0.00931	10656	0.00837	4.6
Upregulated Aging Genes	Gfi	Zinc-coordinating	BetaBetaAlpha-zinc finger	132	105	8480	4397	550	0.0279	40098	0.0262	4.5
Upregulated Aging Genes	Ddit3::Cebpa	Zipper-Type	Leucine Zipper	68	169	4780	8097	125	0.0076	8620	0.00677	4.5
Upregulated Aging Genes	HNF1A	Helix-Turn-Helix	Homeo	30	207	2090	10787	41	0.00291	2631	0.00241	4.5
Upregulated Aging Genes	CREB1	Zipper-Type	Leucine Zipper	75	162	5388	7489	153	0.0062	10435	0.00546	4.4
Upregulated Aging Genes	AP1	Zipper-Type	Leucine Zipper	143	94	8894	3983	679	0.0241	49419	0.0226	4.3
Upregulated Aging Genes	Foxq1	Winged Helix-Turn-Heli	Forkhead	98	139	6701	6176	254	0.0142	18136	0.0131	4.3
Upregulated Aging Genes	SOX9	Other Alpha-Helix	High Mobility Group	119	118	8007	4870	428	0.0195	30976	0.0182	4.2
Upregulated Aging Genes	Sox5	Other Alpha-Helix	High Mobility Group	134	103	8981	3896	755	0.0268	55223	0.0253	4.2
Upregulated Aging Genes	Gsc	Helix-Turn-Helix	Homeo	126	111	8120	4757	413	0.0126	29447	0.0116	4.1
Upregulated Aging Genes	CEBPA	Zipper-Type	Leucine Zipper	143	94	9016	3861	707	0.0323	52050	0.0307	4.1
Upregulated Aging Genes	vnd	Helix-Turn-Helix	Homeo	113	124	7447	5430	357	0.0163	25759	0.0152	4.0
Upregulated Aging Genes	oc	Helix-Turn-Helix	Homeo	120	117	7831	5046	364	0.0111	25900	0.0102	4.0
Downregulated Aging Genes	Esrrb	Zinc-coordinating	Hormone-nuclear Receptor	109	80	4804	8073	238	0.00794	8841	0.00694	7.2
Downregulated Aging Genes	MEF2A	Other Alpha-Helix	MADS	103	86	5047	7830	266	0.00739	9936	0.0065	6.6
Downregulated Aging Genes	gt	Zipper-type	Leucine Zipper	71	118	2980	9897	115	0.0032	4135	0.00271	5.6
Downregulated Aging Genes	hth	Helix-Turn-Helix	Homeo	148	41	8223	4654	927	0.0155	36588	0.0144	5.5
Downregulated Aging Genes	Pax6	Helix-Turn-Helix	Homeo	34	155	1182	11695	40	0.00156	1350	0.00124	5.4
Downregulated Aging Genes	HOXA5	Helix-Turn-Helix	Homeo	169	20	10289	2588	3644	0.081	150368	0.0787	5.1
Downregulated Aging Genes	HLF	Zipper-Type	Leucine Zipper	113	76	5284	7593	260	0.00867	10110	0.00794	4.9
Downregulated Aging Genes	vis	Helix-Turn-Helix	Homeo	146	43	7694	5183	756	0.0126	29848	0.0117	4.9
Downregulated Aging Genes	PBX1	Helix-Turn-Helix	Homeo	89	100	4013	8864	177	0.0059	6767	0.00532	4.8
Downregulated Aging Genes	run::Bgb	Ig-fold	Runt	121	68	6193	6684	380	0.00951	14864	0.00876	4.8
Downregulated Aging Genes	RREB1	Zinc-coordinating	BetaBetaAlpha-zinc finger	17	172	771	12106	25	0.00139	859	0.00112	4.7
Downregulated Aging Genes	Sox17	Other Alpha-Helix	High Mobility Group	155	34	8674	4203	1138	0.0285	46334	0.0273	4.3
Downregulated Aging Genes	achi	Helix-Turn-Helix	Homeo	150	39	8011	4866	849	0.0142	33978	0.0133	4.3
Downregulated Aging Genes	z	Helix-Turn-Helix	Zeste	145	44	7862	5015	761	0.0212	30843	0.0202	4.1
Downregulated Aging Genes	FOXI1	Winged Helix-Turn-Heli	Forkhead	152	37	8378	4499	960	0.032	39276	0.0309	4.1
