# Supplementary material for: Expression patterns of cardiac aging in Drosophila
Source: Aging Cell. 2017 Jan 16;16(1):82–92. doi: 10.1111/acel.12559 (PMC5242310; doi:10.1111/acel.12559)
Supplement: Supplementary file 8 [file ACEL-16-82-s008.pdf]

List of supporting information

SUPPLEMENTARY TABLES

Supplementary Table 1: Age-related genes

Supplementary Table 2: GO terms enriched in up- and down-regulated genes

Supplementary Table 3: Genes differentially regulated in fly and rodent gene sets

Supplementary Table 4: oPOSSUM transcription factor binding site enrichment analysis in proximal promoters of aging responsive genes.

Supplementary Figure 1. Heart-to-heart variability in normalized gene expression. Hearts from 1- and 5-week old *yw* flies. n=15-16. Permutation test p<0.05. Gene expression normalized to expression of *Mcm2*.

Supplemental Figure 2. Induced *Odd* expression in old flies. A) 5-6-week-old female *Drosophila* hearts labeled for *Odd* (green) and Gapdh1 (red) transcripts. Shown are 5 week old female hearts, with heart-specific expression of RU486-inducibed Gal4 (Hand-GS-Gal4). Under RU486-free baseline conditions (left three panels), *Odd* expression is detected in pericardial cells (PCs, arrowheads), and lower levels in the heart tube (outlined area). Induction of Gal4 activity causes up-regulation of *Odd* RNA in the heart tube and PCs. B) Quantification of *Odd* transcript induction following RU486-administration for 1 week (*p<0.05, unpaired t-test).

Supplemental Figure 3. miR-1 expression. A) qPCR of miR-1 expression in 1- and 5-week-old fly hearts. B) miR-1 sequence alignment showing conservation of sequence between *D. melanogaster* and human (hsa), and *D. melanogaster* and mouse (mmu). Sequences from miRBase (http://www.mirbase.org).
